# Culture eats evidence for breakfast: how culture influences implementation of evidence-based practices

**DOI:** 10.1186/s43058-026-00940-z

**Published:** 2026-04-29

**Authors:** Per Nilsen, Jeanette Wassar Kirk

**Affiliations:** 1 Department of Health, Medicine and Caring Sciences, Linköping University, Linköping, Sweden; 2 Faculty of Medicine and Health Sciences, Halmstad University, Halmstad, Sweden; 3 Department of Clinical Research, Hvidovre University Hospital, Copenhagen, Denmark; 4 Department of Health and Social Context, National Institute of Public Health, University of Southern Denmark, Odense, Denmark

**Keywords:** Culture, Evidence, Strategies, Profession, Discipline, Context, Mechanism

## Abstract

**Background:**

Research has produced a substantial and expanding body of evidence-based practices (EBPs), encompassing interventions, programmes, clinical guidelines, protocols, care pathways and models of care supported by the best available evidence. Despite this, healthcare delivery is still frequently characterised as insufficiently evidence-based, reflecting a persistent gap between what is known to be effective and what is routinely implemented in practice. Traditional explanations only partially account for this gap, often overlooking culture as a critical yet under-theorized influence. Culture is a learned phenomenon rooted in social contexts, encompassing shared norms, values, beliefs and assumptions that define a group, whether a team, profession or organization. This paper argues that the uptake and sustainability of EBPs are profoundly shaped by cultural dynamics operating across three key layers: organizational, professional and disciplinary.

**Main body:**

Organizational culture shapes openness to change, learning and psychological safety, influencing whether EBPs are seen as improvements or burdens. Professional cultures, rooted in education and identity, affect how physicians, nurses and other professionals apply guidelines and protocols. Disciplinary cultures, tied to clinical environments (e.g. emergency, intensive, mental health, palliative care), also shape how EBPs are received. Enhancing cultural responsiveness requires aligning EBPs with the shared norms, values, beliefs and assumptions of the intended users. Strategies include fostering clinician engagement in the development of EBPs, cultural competence, local adaptation and leveraging cultural champions.

**Conclusion:**

Implementation of EBPs is shaped by culture, not solely by the strength of evidence or implementation strategies. Organizational, professional and disciplinary cultures interact to influence how EBPs are interpreted, accepted, adapted or resisted in practice, helping to explain persistent variation in uptake. Misalignment between EBPs and prevailing norms, values, beliefs and assumptions undermines implementation even when evidence is strong.

Contributions to the literature

- Reframes implementation of EBPs as a fundamentally cultural process, rather than a primarily technical or procedural one, emphasizing the role of organizational, professional and disciplinary cultures.
- Highlights how misalignment between EBPs and culture undermines uptake, even with strong evidence.
- Advocates for culturally responsive implementation strategies.
- Proposes concrete, culturally responsive approaches to enhance cultural understanding in implementation strategies.

## Introduction

Research has produced a substantial and expanding body of evidence-based practices (EBPs), encompassing interventions, programmes, clinical guidelines, protocols, care pathways and models of care supported by the best available evidence. These EBPs are intended to inform clinical decision-making, enhance quality and safety, and reduce unwarranted variation in care. Nevertheless, healthcare delivery continues to be characterised as insufficiently evidence-based, reflecting a persistent gap between what is known to be effective and what is routinely enacted in practice [1–5].

Robust evidence alone does not ensure uptake or sustained use. Addressing this gap requires implementation, understood as the deliberate and systematic integration of EBPs into routine practice within specific settings, as distinct from diffusion, which occurs more passively and informally through unstructured spread [1, 2, 4, 5]. Implementation commonly depends on active and sustained strategies, including training, facilitation, audit and feedback, workflow redesign, leadership engagement and alignment with existing systems and incentives. However, even carefully selected implementation strategies may fail if they do not adequately account for the social and organizational contexts in which EBPs are introduced [1, 2, 4, 5].

As a result, EBPs are often ignored, superficially applied or actively resisted in practice [1–5]. Explanations for this persistent evidence-practice gap typically focus on factors such as time pressure, staff shortages, limited resources and ineffective implementation strategies [1, 6]. While important, these accounts tend to overlook a deeper and potentially more decisive factor: culture.

Definitions of culture vary, but most scholars agree it is a learned phenomenon rooted in social contexts, encompassing shared norms, values, beliefs and assumptions that define a group, whether a team, profession or organization [7–9]. Norms refer to informal rules about acceptable behaviour; values are the core ideals about what is important; beliefs are perceived truths that shape how information is interpreted; and assumptions are deeply embedded, often unconscious ideas that influence meaning-making and action [10, 11]. As a fundamentally collective phenomenon, culture frequently outweighs individual differences, shaping cognition, emotion and behaviour [12]. Culture is not always visible or consciously acknowledged [12].

Humans are biologically and psychologically adapted to group living, making us inherently social beings whose thoughts and actions are shaped by social context. Our evolutionary success has depended on cooperation, imitation and social cohesion [13, 14]. Culture, transmitted through language and imitation, enables knowledge to accumulate beyond individual capacity [15, 16]. Classic studies, including Asch’s [17] conformity experiments and social identity theory [18], elucidate how individuals adopt group norms and values, even over personal judgment, to maintain group inclusion. The theory of situational strength [19] further suggests that behaviour is shaped most by external cues in “strong” situations, where group pressure is clearly signalled. In “weak” situations, where such cues are absent or ambiguous, individual traits play a greater role. These findings underscore that culture is an important lens for understanding the human condition.

In the context of implementation science, culture should not be viewed as a passive backdrop or a variable to be “controlled” because culture actively shapes how guidelines and protocols are interpreted, accepted and sustained. Implementing EBPs is not about having the “right” culture in place, but about how healthcare staff, leaders and systems co-create cultural conditions that enable or hinder implementation. Culture is embedded in routines, reinforced by hierarchies and expressed through professional identities [20, 21]. It shapes what counts as legitimate knowledge, appropriate actions, who can question norms and how power functions within teams, professions and organizations [12, 22, 23]. These dynamics directly influence how EBPs are understood and integrated into daily practice [24–27]. Culture is inseparable from power. Norms, values, beliefs and assumptions do not simply emerge; they are shaped, reinforced and contested through professional hierarchies, regulatory frameworks and institutional authority. Epistemic authority, i.e. who determines what counts as legitimate knowledge, is central to EBP implementation. Cultural receptivity to EBPs is therefore not only a matter of shared meaning, but also of whose interpretations prevail.

Yet despite its omnipresence, culture remains under-theorized in implementation science. Although organizational culture features in numerous frameworks within the field [6], cultural influences operating at other levels, such as professional or disciplinary, are rarely examined explicitly. As a result, healthcare environments are often implicitly treated as neutral or passive recipients of change, in which strong evidence and well-designed implementation strategies are assumed to be sufficient to produce uptake [28–30]. This assumption overlooks the culture through which EBPs must travel. Implementation strategies that ignore culture risk looking good on paper but falling flat in practice.

This article takes a clear position: implementation science must put culture at the centre of analysis to understand why implementation of EBPs succeeds or fails. Drawing on three intersecting cultural layers (organizational, professional and disciplinary), this paper argues that how EBPs are interpreted, received and applied in practice depends on their alignment with the cultural realities of those expected to use them.

Broader cultural influences also exist, including regional, generational, ethnic and national cultures, and these may shape healthcare practices in important ways. However, the focus of this paper is deliberately narrowed to cultural layers that are most proximal to everyday implementation work and most directly implicated in the implementation of EBPs into routine healthcare practice. By concentrating on organizational, professional and disciplinary cultures, the analysis targets cultural dynamics that are embedded in clinical roles, institutional norms, training traditions and knowledge hierarchies, factors that implementation strategies can realistically engage with and seek to influence. These layers should be understood as analytic distinctions rather than bounded entities, as they are likely interdependent.

The professional and disciplinary distinctions presented in this paper should be understood as analytic heuristics rather than fixed or homogeneous categories. Substantial intra-professional variation exists across specialties, institutional settings, career stages and national contexts. The cultural patterns described here are not intended as essentialized traits of professions or disciplines, but as recurring tendencies documented in empirical studies. Recognizing heterogeneity and internal contestation is crucial to avoid reifying culture as static or uniform.

The aim is to explore how organizational, professional and disciplinary cultural layers shape the implementation of EBPs such as clinical guidelines and standardized protocols, demonstrating that meaningful implementation requires more than strong evidence and well-designed strategies; it also requires cultural alignment.

The title of this paper is not intended to suggest a dichotomy or competition between evidence and culture, nor to imply that culture should replace or outweigh scientific evidence. Rather, it is used deliberately to underscore the central importance of culture in shaping how evidence, regardless of its methodological rigour or strength, is interpreted, valued and acted upon in practice. In this sense, the title highlights culture as a foundational condition for implementation, one that can enable or constrain the uptake of EBPs irrespective of the quality of the evidence itself.

### Organizational cultures

Organizational culture refers to the shared norms, values, beliefs and assumptions within an organization such as a healthcare organization [10, 31]. It influences everything from communication styles to leadership dynamics and plays a critical role in shaping how employees respond to change [32–34]. In healthcare organizations, this culture is not merely reflected in formal policies or mission statements but exists in the everyday routines, informal interactions and unspoken assumptions that shape how care is delivered [35]. It shapes what is prioritized, how decisions are made and how open staff and managers are to new knowledge and practices [12]. These characteristics indicate that organizational culture plays a significant role in determining how successfully EBPs can be implemented [31, 35].

The success of implementation of EBPs can vary widely depending on an organization’s culture. Organizations that cultivate a just culture, emphasizing learning over blame, promoting fair accountability and ensuring psychological safety, are generally more adaptable and receptive to the implementation of EBPs [36–38]. In these environments, staff are encouraged to ask questions, challenge outdated practices and collaborate across disciplines. Mistakes are viewed as learning opportunities rather than failures, which cultivates a culture where continuous improvement is possible [12, 39]. EBPs are more likely to be embraced to enhance care quality, not just as checklist items or external requirements [40–42].

On the other hand, organizations with a blame culture, rigid hierarchies or chronic resource shortages may resist change, even when EBPs are backed by strong evidence and effective strategies [43–45]. In such contexts, new guidelines or protocols can feel disconnected from the realities of daily care, perceived as top-down mandates rather than meaningful tools to improve care [46, 47]. Staff may be reluctant to speak up or propose changes, fearing reprimand or lack of support. As a result, EBPs are often implemented superficially, aimed more at satisfying audit requirements than transforming practice [48, 49].

### Professional cultures

Professional identities in healthcare are deeply rooted and give rise to distinct cultures within and across disciplines. These identities are shaped over time through formal education, clinical training and the everyday socialization that occurs within peer groups [50, 51]. As a result, professional silos often form, potentially hindering the collaboration needed to deliver optimal care [52–54]. Professional cultures influence not only how clinicians see their roles and responsibilities but also how they define quality care and make clinical decisions [50, 51, 55, 56]. Thus, professional cultures can play an important role in either enabling or obstructing the implementation of EBPs.

In many contexts, physicians are trained within institutionalized professional traditions that emphasize autonomy, clinical judgement and decision-making authority. Empirical studies have documented patterns in which physicians’ professional identities are shaped by expectations to lead, deliver definitive diagnoses and take responsibility for complex clinical decisions [57–59]. EBPs may be more readily accepted when they are perceived to complement individual expertise and support, rather than constrain, clinical discretion. Clinical guidelines or standardized protocols perceived as overly prescriptive or lacking flexibility may be resisted if they are seen to undermine the physician’s role as an independent decision-maker [60, 61].

In many healthcare settings, nurses are socialized within professional traditions that value teamwork, coordination, patient advocacy and adherence to established care processes [55, 62]. Empirical literature has identified institutionalized norms within segments of nursing that emphasize consistency, safety and holistic care, potentially creating a more receptive context for clinical guidelines and standardized protocols, particularly when these align with patient-centred values. However, rigid EBPs that overlook the relational, emotional and situational dimensions of nursing practice may be met with scepticism or informally adapted if they fail to support the clinical judgement that nurses are trained to exercise in bedside care [63–65].

Even within the broader professions of medicine and nursing, substantial cultural differences exist between medical specialties (e.g. surgeons and general practitioners) and among the diverse roles within nursing (e.g. registered nurses and nurse assistants). A guideline or protocol may be readily embraced in one specialty but resisted in another, even within the same professional domain, depending on how well it aligns with the specific priorities, routines and day-to-day realities of those expected to use the EBPs [66].

Empirical studies of allied health professionals, including physiotherapists and occupational therapists, have documented orientations toward long-term functional outcomes, rehabilitation goals and individualized assessments. In many contexts, practice within these professions spans multiple sessions and environments, requiring ongoing evaluation and adaptation [67–69]. Accordingly, EBPs may be better aligned when they support flexible, goal-oriented care and accommodate longitudinal clinical reasoning. Tools that emphasize short-term metrics or acute outcomes may be perceived as misaligned with the broader goals of these professions [70, 71].

These distinctions should not obscure substantial variation within professions. Specialty-specific norms, institutional contexts, resource conditions and generational differences may produce markedly different cultural orientations within the same professional category. Understanding implementation therefore requires attention to how professional identities are locally enacted rather than assumed.

### Disciplinary cultures

Another important cultural layer within hospitals and healthcare organizations arises from clinical disciplines and specific care environments. These cultures are shaped by the nature of clinical work, the pace and intensity of activity, hierarchical structures and patterns of professional interaction [7, 72]. Such variations are especially pronounced in environments such as emergency departments, intensive care units, palliative care and mental health services. In such environments, disciplinary cultures shape communication practices, decision-making processes and approaches to care delivery, all of which influence the implementation of EBPs [6, 34, 35, 72].

In emergency care environments, the culture is fast-paced, high-stake and inherently unpredictable. It demands rapid responses from multidisciplinary teams operating under intense pressure and time constraints. Clinicians are often required to make split-second decisions based on limited information, emphasizing the need for swift, direct communication. A relatively flat team hierarchy supports agile collaboration, enabling faster coordination during critical moments [73]. However, the constant urgency and emotional intensity of this environment can contribute to high staff turnover, emotional exhaustion and burnout [74, 75]. In such contexts, EBPs are more readily accepted when they are actionable and tailored to support rapid clinical decision-making. Conversely, guidelines that are overly lengthy, complex or rigid may face resistance if they are perceived as slowing down care or limiting adaptability [76].

Similarly, intensive care units operate under highly technical, detail-oriented demands where clinical vigilance is essential. Nurses often serve as frontline interpreters of patient status, exercising critical thinking and initiating proactive interventions [77–79]. Care is delivered by multidisciplinary teams, with nurses often playing a pivotal role as patient advocates, drawing on their expertise to detect and respond to early signs of deterioration [26, 27]. In this context, EBPs that promote safety and support vigilance are generally well received [80, 81]. However, tools that are overly complex, time-consuming or poorly aligned with intensive care workflows may be rejected, particularly during high-pressure situations such as patient admissions. Effective implementation, therefore, hinges on integration into existing routines, minimizing cognitive load and procedural disruption while enhancing critical care delivery [82].

Palliative care shifts the focus from cure to comfort, emphasizing dignity, compassion and quality of life. It is inherently multidisciplinary, grounded in a holistic, person-centred approach that seeks to alleviate the emotional, psychological, physical, social and spiritual distress experienced by individuals with serious illness and their families. Core team members represent a range of professions, each contributing unique expertise to support patients through serious illness or end-of-life care. The slower pace of palliative care allows for deeper, more empathetic communication, and the interdisciplinary team places equal value on emotional and spiritual well-being as on physical health [83, 84]. Within this environment, EBPs are more readily embraced when they reinforce individualized, humane care and align with the values of person-centred practice. In contrast, rigid or overly complex guidelines and protocols may be less effective unless adapted to the specific needs of patients and families [85–87].

In mental health care, the prevailing culture is deeply rooted in interpersonal connection and trust, which are foundational to effective treatment and recovery. Care is delivered by multidisciplinary teams typically comprising psychiatrists, psychologists, social workers, nurses and other professionals, each contributing specialized expertise to address complex emotional and psychological needs. Staff are trained to respond sensitively to emotional distress and to cultivate therapeutic relationships that foster safety, engagement and healing. Relational presence is often prioritized alongside procedural tasks, reflecting the central role of human connection in mental health recovery [88, 89]. EBPs are more likely to be accepted when they support these relational dynamics and allow for clinical discretion and individualized care. Conversely, tools that are seen as oversimplifying the complexity of mental health or that conflict with the humanistic values and ethical principles of the discipline may be met with resistance or scepticism [90–92].

These disciplinary depictions represent stylized accounts intended to illuminate how work conditions shape cultural patterns. Emergency departments, intensive care units or mental health services may differ significantly across countries, institutions and resource contexts. Culture within disciplines is therefore better understood as emergent and situational rather than uniform or deterministic.

### Operationalizing cultural analysis in implementation

Implementation is more likely to succeed when EBPs align with the shared norms, values, beliefs and assumptions of those expected to use them. Operationalizing cultural analysis in implementation therefore requires systematic attention to how these shared meanings are produced, sustained and enacted within organizational and professional contexts.

Cultural competence can be understood not only as an individual attribute but as a collective capacity embedded in professional education and continuous development. Such capacity enables clinicians and other health professionals to navigate the cultural diversity of organizations, professions and disciplines, positioning them not merely as users of guidelines and protocols but as interpreters who mediate between formal evidence and the realities of everyday care [93]. In this sense, training functions as a site where professional identities, norms and interpretive frameworks are shaped and potentially recalibrated.

Cultural analysis also entails examining the context into which EBPs are introduced. Assessing cultural conditions prior to implementation can help identify the shared norms, values, beliefs and assumptions that influence how guidelines and protocols are interpreted and enacted [12, 94]. Because many cultural elements are tacit and difficult to recognize from within, adopting an external or reflexive analytical stance may help surface taken-for-granted assumptions and implicit hierarchies [12].

Ensuring cultural responsiveness further requires attention to how implementation processes interact with existing relationships, authority structures and informal influence networks. Involving frontline staff and leadership in interpreting and adapting EBPs can be understood as a mechanism through which legitimacy is negotiated and contextual relevance established. Similarly, so-called cultural champions, i.e. respected and influential staff who support change, illustrate how peer authority and informal norms shape uptake [94–96]. Cultural transformation, however, unfolds gradually, as shifts in meaning and legitimacy become embedded in routines, relationships and everyday practice rather than occurring through discrete interventions alone [46, 91, 97, 98].

## Discussion

The successful implementation of EBPs such as interventions, programmes, clinical guidelines, protocols, care pathways and models of care supported by the best available evidence is shaped by multiple layers of culture, including organizational, professional and disciplinary cultures. Aside from broader cultural influences, such as regional, generational, ethnic and national cultures, distinct subcultures within organizations can also shape how EBPs are applied in practice, e.g. learning culture [99], innovation culture [100], patient safety culture [101] and quality culture [102]. Communities of practice also constitute an important cultural layer, shaping how EBPs are interpreted, negotiated and enacted through everyday social interaction [50]. These and other cultural layers interact in complex and often overlapping ways, jointly influencing whether EBPs are embraced, adapted or resisted in practice. Rather than operating as discrete or hierarchical factors, culture is inherently multidimensional, with multiple intersecting layers simultaneously shaping how EBPs are understood and taken up.

Implementation science research must grapple with the reality that EBPs are enacted within settings characterised by multiple, intersecting cultures. Organizational, professional and disciplinary cultures may be internally diverse and are dynamically produced through social interaction, rather than being fixed or uniform. Attending to this potential heterogeneity is essential for understanding implementation as a socio-technical process embedded in both local practices and wider institutional contexts.

Resistance should not be reduced to cultural inertia. It may represent rational clinical judgment, particularly where guidelines are perceived as poorly adapted to patient complexity. It may also reflect organized defense of professional autonomy or institutional interests. Viewing resistance as agency rather than barrier reframes implementation as negotiated practice rather than linear adoption.

Although organizational culture has been studied extensively in organizational research and to a lesser extent in implementation science [31, 103], much less attention has been paid to cultural dynamics at the professional and disciplinary levels in the context of implementing EBPs. This distinction can be clarified through engagement with Normalisation Process Theory (NPT). Within this framework, social structure refers to the relational and material conditions that shape and constrain action in practice, including formal roles, distributions of expertise, access to organizational and technological resources, and regulatory or managerial constraints [104, 105]. Social structure thus concerns the patterned arrangements of authority, responsibility, and resources that make certain actions possible and others difficult. By contrast, culture refers to shared meanings, legitimacy claims, taken-for-granted assumptions, and normative understandings of what constitutes appropriate or valuable practice. Culture shapes how actors interpret interventions, how they define professional identity, and how they evaluate whether a new practice “fits” within their understanding of good care. While NPT provides a robust account of the structural and interactional mechanisms through which practices become embedded, it does not in itself constitute a theory of culture; rather, cultural analysis adds a complementary focus on shared meaning, legitimacy, and taken-for-granted assumptions.

Although analytically distinct, culture and social structure are dynamically related through interaction. Social structures enable certain cultural norms and values to persist by institutionalizing them in roles, workflows, and incentive systems; at the same time, cultural beliefs and assumptions legitimize and reproduce particular structural arrangements. For example, professional hierarchies (structure) may sustain taken-for-granted beliefs about epistemic authority (culture), while regulatory mandates (structure) may reconfigure what is considered legitimate practice (culture). Empirically, this distinction enhances methodological clarity. Researchers might operationalize culture through analysis of narratives, informal norms and values, professional identity claims, and everyday accounts of legitimacy and appropriateness. In contrast, social structure may be examined through formal authority arrangements, documented role definitions, resource allocation patterns, accountability mechanisms, and incentive systems. Interactional processes, which are central to NPT, provide the observable arena in which structural conditions and cultural meanings are enacted and mutually reinforced. In this way, NPT offers an analytical lens for examining how implementation unfolds within structured conditions of action, complementing rather than collapsing into cultural analysis.

Cultural norms and values are sustained not only through shared meaning but through institutionalized power relations. Professional groups differ in epistemic authority, resource control and decision-making autonomy. Resistance to EBPs may therefore reflect strategic defense of jurisdictional boundaries rather than cultural misalignment alone. Similarly, external actors such as regulators, payers and accreditation bodies shape the incentive structures within which EBPs are introduced. Cultural receptivity cannot be understood independently of these structural interests. Culture is thus both shaped by and productive of power. A power-sensitive cultural lens allows implementation scholars to examine not only whether EBPs align with norms and values, but whose norms and values prevail and whose interests are advanced.

Despite its importance, culture is notoriously difficult to assess analytically because its deepest elements are often unconscious. As argued by Schein [12], culture tends to be taken for granted, making it difficult for insiders to identify its defining elements and rendering them effectively “culture blind”. Schein advocates for the use of external perspectives or an anthropological stance: stepping back to observe, reflect critically and question what typically goes unquestioned.

This analytical difficulty may partly explain why previous efforts to foreground culture in healthcare reform have struggled, as culture was often treated as a monolithic variable rather than as layered, contested and embedded in power relations. By differentiating organizational, professional and disciplinary layers, and situating culture within structural and epistemic dynamics, this paper seeks to refine rather than romanticize the concept.

Another challenge is to identify which cultural layer exerts the greatest influence on practice. Organizational, professional and disciplinary cultures can overlap and interact in subtle, dynamic ways. For example, a physician working in palliative care may be trained within the professional culture of medicine, which traditionally emphasizes a biomedical model focused on diagnosis and treatment. However, the disciplinary culture of palliative care prioritizes holistic, team-based approaches that involve emotional, psychosocial and spiritual support. This tension underscores the challenge clinicians face in navigating and reconciling competing expectations, prompting the question of which cultural layer ultimately holds the greatest influence over practice.

This paper places culture as central to implementation success, but it is important to recognize that it is not always the decisive factor. In some contexts, structural constraints, such as staff shortages, limited resources or regulatory pressures, can directly affect the feasibility of implementing EBPs. However, the COVID-19 pandemic revealed that rapid implementation of new routines, such as digitally enabled remote counselling and reconfigured patient care pathways, was not driven by urgency or top-down directives alone, but by the presence of flexible, resilient workplace cultures that were open to adaptation [106–108]. These environments were better able to respond under pressure because cultures oriented towards flexibility and responsiveness can actively facilitate change [6, 24].

In this context, the phrase “culture eats evidence for breakfast” takes on renewed significance, not as a critique of weak evidence or flawed strategies but as recognition that culture fundamentally shapes how change is perceived and enacted. Even the most rigorously developed guidelines and protocols will falter if they are misaligned with the shared norms, values, beliefs and assumptions among those expected to adopt them. Implementation science must move beyond viewing culture as a passive backdrop or a mere barrier to change, and instead recognize it as a dynamic force that can be understood as context, mechanism and outcome. Only by doing so can we begin to close the persistent gap between evidence and practice in healthcare. Until then, even the strongest evidence will remain just that, evidence, while culture continues to shape what happens where it matters most: at the point of care.

In conclusion, this paper argues that the implementation of EBPs is shaped by culture, not solely by the strength of evidence or implementation strategies. Organizational, professional and disciplinary cultures interact to influence how EBPs are interpreted, accepted, adapted or resisted in practice, helping to explain persistent variation in uptake. Misalignment between EBPs and prevailing norms, values, beliefs and assumptions undermines implementation even when evidence is strong.

## Data Availability

Not applicable.

## References

[1] Palinkas LA. Achieving Implementation and Exchange: The Science of Delivering Evidence-Based Practices to At-Risk Youth. Policy Press; 2018. 10.56687/9781447338147.

[2] Hoffmann T, Del Mar C, Bennett S. Evidence-based practice across the health professions. Chatswood, N.S.W.: Churchill Livingstone/Elsevier; 2010.

[3] Sadeghi-Bazargani H, Tabrizi JS, Azami-Aghdash S. Barriers to evidence-based medicine: a systematic review. Evaluation Clinical Practice. 2014;20:793–802. 10.1111/jep.12222. 10.1111/jep.12222 25130323

[4] Gray JAM, Shepperd S. Evidence-based healthcare and public health: how to make decisions about health services and public health. 3. ed. reprinted. Edinburgh: Churchill Livingstone; 2010.

[5] Ellis P. Evidence-based practice in nursing. 4th edition. London Thousand Oaks, CA New Delhi Singapore: SAGE Learning Matters; 2019.

[6] Nilsen P. Implementation Science: Theory and Application. 1st ed. London: Routledge; 2024. 10.4324/9781003318125.

[7] Schein EH. The role of the founder in creating organizational culture. Organ Dyn. 1983. 10.1016/0090-2616(83)90023-2.

[8] Geertz C. The Interpretation of Cultures. New York: Basic Books; 1973.

[9] Greenhalgh T, Robert G, Bate P, Macfarlane F, Kyriakidou O. Diffusion of Innovations in Health Service Organisations : A Systematic Literature Review. 1st ed. Hoboken: Wiley; 2008.

[10] Bang H. Organisationskultur. 2. uppl. Lund: Studentlitteratur; 1999.

[11] Grimm NA, Lee SK. How do organizational culture and strategy influence implementation of evidence-based practice? AMIA Annu Symp Proc. 2005;2005:970. 16779257 PMC1560500

[12] Schein EH. Organizational culture and leadership. 4th ed. San Francisco: Jossey-Bass; 2010.

[13] Dunbar RIM. Neocortex size as a constraint on group size in primates. J Hum Evol. 1992;22:469–93. 10.1016/0047-2484(92)90081-J.

[14] Rizzolatti G, Craighero L. THE MIRROR-NEURON SYSTEM. Annu Rev Neurosci. 2004;27:169–92. 10.1146/annurev.neuro.27.070203.144230. 15217330 10.1146/annurev.neuro.27.070203.144230

[15] Boyd R, Richerson PJ. Culture and the evolutionary process. Paperback ed. Chicago: University of Chicago Press; 1988.

[16] Henrich J. The Secret of Our Success: How Culture Is Driving Human Evolution, Domesticating Our Species, and Making Us Smarter. Princeton University Press; 2015. 10.2307/j.ctvc77f0d.

[17] Asch SE. Studies of independence and conformity: I. A minority of one against a unanimous majority. Psychol Monogr Gen Appl. 1956;70:1–70. 10.1037/h0093718.

[18] Tajfel H, Turner JC. An integrative theory of intergroup conflict. In: Austin WG, Worchel S, editors. The Social Psychology of Intergroup Relations Monterey. CA: Brooks/Cole; 1979. p. 33–47.

[19] Meyer RD, Dalal RS, Hermida R. A review and synthesis of situational strength in the organizational sciences. J Manage. 2010;36:121–40. 10.1177/0149206309349309.

[20] Kirk JW, Nilsen P. Implementing evidence-based practices in an emergency department: contradictions exposed when prioritising a flow culture. J Clin Nurs. 2016;25(3-4):555-65. 10.1111/jocn.13092. 10.1111/jocn.13092 PMC4738684 26818380

[21] Essex R, Kennedy J, Miller D, Jameson J. A scoping review exploring the impact and negotiation of hierarchy in healthcare organisations. Nurs Inq. 2023;30:e12571. 10.1111/nin.12571. 37338510 10.1111/nin.12571

[22] Hofstede G, Hofstede GJ, Minkov M. Kulturer og organisationer: overlevelse i en grænseoverskridende verden. 3. udgave. Kbh: Handelshøjskolens Forlag; 2021.

[23] Coolen PR. Cultural relevance in end-of-life care. New York, NY: Springer Publishing Company; 2012.

[24] Dodek P, Cahill NE, Heyland DK. The relationship between organizational culture and implementation of clinical practice guidelines: a narrative review. J Parenter Enteral Nutr. 2010;34:669–74. 10.1177/0148607110361905. 10.1177/0148607110361905 21097767

[25] Li S-A, Jeffs L, Barwick M, Stevens B. Organizational contextual features that influence the implementation of evidence-based practices across healthcare settings: a systematic integrative review. Syst Rev. 2018;7:72. 10.1186/s13643-018-0734-5. 29729669 10.1186/s13643-018-0734-5 PMC5936626

[26] Mannion R, Davies H. Understanding organisational culture for healthcare quality improvement. BMJ 2018:k4907. 10.1136/bmj.k4907. 10.1136/bmj.k4907 PMC6260242 30487286

[27] Mbau R, Gilson L. Influence of organisational culture on the implementation of health sector reforms in low- and middle-income countries: a qualitative interpretive review. Glob Health Action. 2018;11:1462579. 10.1080/16549716.2018.1462579. 29747550 10.1080/16549716.2018.1462579 PMC5954479

[28] Cassidy CE, Harrison MB, Godfrey C, Nincic V, Khan PA, Oakley P, et al. Use and effects of implementation strategies for practice guidelines in nursing: a systematic review. Implement Sci. 2021;16:102. 10.1186/s13012-021-01165-5. 34863220 10.1186/s13012-021-01165-5 PMC8642950

[29] Tietschert M, Bahadurzada H, Kerrissey M. Revisiting organizational culture in healthcare: heterogeneity as a resource. Soc Sci Med. 2024;356:117165. 10.1016/j.socscimed.2024.117165. 39121526 10.1016/j.socscimed.2024.117165

[30] Gertner AK, Franklin J, Roth I, Cruden GH, Haley AD, Finley EP, et al. A scoping review of the use of ethnographic approaches in implementation research and recommendations for reporting. Implementation Research and Practice. 2021;2:263348952199274. 10.1177/2633489521992743. 10.1177/2633489521992743 PMC8153409 34056611

[31] Scott T. Implementing culture change in health care: theory and practice. Int J Qual Health Care. 2003;15:111–8. 10.1093/intqhc/mzg021. 12705704 10.1093/intqhc/mzg021

[32] Hung DY, Leidig R, Shelley DR. What’s in a setting?: influence of organizational culture on provider adherence to clinical guidelines for treating tobacco use. Health Care Manage Rev. 2014;39:154–63. 10.1097/HMR.0b013e3182914d11. 23636103 10.1097/HMR.0b013e3182914d11

[33] Ukawa N, Tanaka M, Morishima T, Imanaka Y. Organizational culture affecting quality of care: guideline adherence in perioperative antibiotic use. Int J Qual Health Care. 2015;27:37–45. 10.1093/intqhc/mzu091. 25502553 10.1093/intqhc/mzu091

[34] Braithwaite J, Herkes J, Ludlow K, Testa L, Lamprell G. Association between organisational and workplace cultures, and patient outcomes: systematic review. BMJ Open. 2017;7:e017708. 10.1136/bmjopen-2017-017708. 29122796 10.1136/bmjopen-2017-017708 PMC5695304

[35] Nutley SM, Walter I, Davies HTO. Using evidence: how research can inform public services. Bristol, U.K.: Policy Press; 2007.

[36] Van Baarle E, Hartman L, Rooijakkers S, Wallenburg I, Weenink J-W, Bal R, et al. Fostering a just culture in healthcare organizations: experiences in practice. BMC Health Serv Res. 2022;22:1035. 10.1186/s12913-022-08418-z. 35964117 10.1186/s12913-022-08418-z PMC9375400

[37] Malik RF, Buljac-Samardžić M, Amajjar I, Hilders CGJM, Scheele F. Open organisational culture: what does it entail? Healthcare stakeholders reaching consensus by means of a Delphi technique. BMJ Open. 2021;11:e045515. 10.1136/bmjopen-2020-045515. 34521658 10.1136/bmjopen-2020-045515 PMC8442051

[38] O’Donovan R, McAuliffe E. Exploring psychological safety in healthcare teams to inform the development of interventions: combining observational, survey and interview data. BMC Health Serv Res. 2020;20:810. 10.1186/s12913-020-05646-z. 32867762 10.1186/s12913-020-05646-z PMC7456753

[39] Wachter RM. Patient Safety At Ten: Unmistakable Progress. Troubling Gaps Health Affairs. 2010;29:165–73. 10.1377/hlthaff.2009.0785. 19952010 10.1377/hlthaff.2009.0785

[40] Edmondson AC. The fearless organization: creating psychological safety in the workplace for learning, innovation, and growth. Hoboken, New Jersey: John Wiley & Sons, Inc; 2019.

[41] Swensen SJ, Pugh MD, McMullan C, Kabcenell A. High-impact leadership: Improve care, improve the health of populations, and reduce costs. Cambridge, MA: Institute for Healthcare Improvement; 2013.

[42] Tucker AL, Edmondson AC. Why Hospitals Don’t Learn from Failures: Organizational and Psychological Dynamics That Inhibit System Change. Calif Manage Rev. 2003;45:55–72. 10.2307/41166165.

[43] Hitchen L. Blame culture is still a problem in tackling patient safety. BMJ. 2007;335:1172.2-1172-a. 10.1136/bmj.39415.492164.DB.

[44] Wolvaardt E. Blame does not keep patients safe. Community Eye Health. 2019;32:36. 31649433 PMC6802475

[45] Yusof ANM, Razali HYH. Moving away from the blame culture: the way forward to manage medical errors. Malays J Med Sci. 2024;31:126–32. 10.21315/mjms2024.31.6.10. 39830101 10.21315/mjms2024.31.6.10 PMC11740813

[46] Dixon-Woods M, Suokas A, Pitchforth E, Tarrant C. An ethnographic study of classifying and accounting for risk at the sharp end of medical wards. Soc Sci Med. 2009;69:362–9. 10.1016/j.socscimed.2009.05.025. 19535193 10.1016/j.socscimed.2009.05.025

[47] Berwick DM. Improvement, trust, and the healthcare workforce. Qual Saf Health Care. 2003. 10.1136/qhc.12.6.448. 14645761 10.1136/qhc.12.6.448 PMC1758027

[48] Francis R. Report of the Mid Staffordshire NHS Foundation Trust Public Inquiry: Executive summary. Presented to Parliament pursuant to Section 26 of the Inquiries Act 2005. London: The Stationery Office; 2013.

[49] Sujan M. An organisation without a memory: a qualitative study of hospital staff perceptions on reporting and organisational learning for patient safety. Reliab Eng Syst Saf. 2015;144:45–52. 10.1016/j.ress.2015.07.011.

[50] Wenger-Trayner É. Communities of practice: learning, meaning, and identity. 18th printing. Cambridge: Cambridge University Press; 2008.

[51] Monrouxe LV. Identity, identification and medical education: why should we care?: Identity, identification and medical education. Med Educ. 2010;44:40–9. 10.1111/j.1365-2923.2009.03440.x. 20078755 10.1111/j.1365-2923.2009.03440.x

[52] Hall P. Interprofessional teamwork: professional cultures as barriers. J Interprof Care. 2005;19:188–96. 10.1080/13561820500081745. 16096155 10.1080/13561820500081745

[53] Dixon-Woods M, Baker R, Charles K, Dawson J, Jerzembek G, Martin G, et al. Culture and behaviour in the English National Health Service: overview of lessons from a large multimethod study. BMJ Qual Saf. 2014;23:106–15. 10.1136/bmjqs-2013-001947. 24019507 10.1136/bmjqs-2013-001947 PMC3913222

[54] Kanjee Z, Bilello L. Leadership & professional development: specialty silos in medicine. J Hosp Med. 2021. 10.12788/jhm.3647. 34129488 10.12788/jhm.3647

[55] Skyvell Nilsson M, Törner M, Pousette A. Professional culture, information security and healthcare quality—an interview study of physicians’ and nurses’ perspectives on value conflicts in the use of electronic medical records. Saf Health. 2018;4:11. 10.1186/s40886-018-0078-9.

[56] Mawuena EK, Mannion R, Adu-Aryee NA, Adzei FA, Amoakwa EK, Twumasi E. Professional disrespect between doctors and nurses: implications for voicing concerns about threats to patient safety. JHOM. 2024;38:1009–25. 10.1108/JHOM-06-2023-0167.

[57] Pype P, Mertens F, Helewaut F, Krystallidou D. Healthcare teams as complex adaptive systems: understanding team behaviour through team members’ perception of interpersonal interaction. BMC Health Serv Res. 2018;18:570. 10.1186/s12913-018-3392-3. 30029638 10.1186/s12913-018-3392-3 PMC6053823

[58] Danielsson M, Nilsen P, Rutberg H, Carlfjord S. The professional culture among physicians in Sweden: potential implications for patient safety. BMC Health Serv Res. 2018;18:543. 10.1186/s12913-018-3328-y. 29996832 10.1186/s12913-018-3328-y PMC6042365

[59] Khawar A, Frederiks F, Nasori M, Mak M, Visser M, Van Etten-Jamaludin F, et al. What are the characteristics of excellent physicians and residents in the clinical workplace? A systematic review. BMJ Open. 2022;12:e065333. 10.1136/bmjopen-2022-065333. 36127103 10.1136/bmjopen-2022-065333 PMC9490566

[60] Cabana MD, Rand CS, Powe NR, Wu AW, Wilson MH, Abboud P-AC, et al. Why don’t physicians follow clinical practice guidelines?: A framework for improvement. JAMA. 1999;282:1458–65. 10535437 10.1001/jama.282.15.1458

[61] Fauman MA. Do physicians use practice guidelines? Psychiatr Times. 2006;23(11).

[62] Mahboube L, Talebi E, Porouhan P, Orak R, Farahani M. Comparing the attitude of doctors and nurses toward factor of collaborative relationships. J Family Med Prim Care. 2019;8:3263. 10.4103/jfmpc.jfmpc_596_19. 31742153 10.4103/jfmpc.jfmpc_596_19 PMC6857374

[63] Allen D. Re-reading nursing and re-writing practice: towards an empirically based reformulation of the nursing mandate. Nurs Inq. 2004;11:271–83. 10.1111/j.1440-1800.2004.00234.x. 15601415 10.1111/j.1440-1800.2004.00234.x

[64] Weller J, Boyd M, Cumin D. Teams, tribes and patient safety: overcoming barriers to effective teamwork in healthcare. Postgrad Med J. 2014;90:149–54. 10.1136/postgradmedj-2012-131168. 24398594 10.1136/postgradmedj-2012-131168

[65] Xu Y, Fan L. Emotional labor and job satisfaction among nurses: the mediating effect of nurse–patient relationship. Front Psychol. 2023;14:1094358. 10.3389/fpsyg.2023.1094358. 37342648 10.3389/fpsyg.2023.1094358 PMC10278545

[66] Cleary J. Nurturing a culture of evidence-based practice: Strategies for implementation in healthcare organizations. J Intensive Crit Care Nurs. 2023;6(4):161.

[67] Lizarondo L, Grimmer K, Kumar S. Assisting allied health in performance evaluation: a systematic review. BMC Health Serv Res. 2014;14:572. 10.1186/s12913-014-0572-7. 25394559 10.1186/s12913-014-0572-7 PMC4234851

[68] Matus J, Wenke R, Hughes I, Mickan S. Evaluation of the research capacity and culture of allied health professionals in a large regional public health service. JMDH. 2019;12:83–96. 10.2147/JMDH.S178696. 30666124 10.2147/JMDH.S178696 PMC6336030

[69] Cordrey T, King E, Pilkington E, Gore K, Gustafson O. Exploring research capacity and culture of allied health professionals: a mixed methods evaluation. BMC Health Serv Res. 2022;22:85. 10.1186/s12913-022-07480-x. 35039018 10.1186/s12913-022-07480-x PMC8764821

[70] Zhang Y, Laber EB, Tsiatis A, Davidian M. Using Decision Lists to Construct Interpretable and Parsimonious Treatment Regimes 2015. 10.48550/ARXIV.1504.07715. 10.1111/biom.12354 PMC4715597 26193819

[71] Iqbal MZ, Rochette A, Mayo NE, Valois M-F, Bussières AE, Ahmed S, et al. Exploring if and how evidence-based practice of occupational and physical therapists evolves over time: a longitudinal mixed methods national study. PLoS ONE. 2023;18:e0283860. 10.1371/journal.pone.0283860. 37000834 10.1371/journal.pone.0283860 PMC10065251

[72] Lok P, Rhodes J, Westwood B. The mediating role of organizational subcultures in health care organizations. J of Health Org and Mgt. 2011;25:506–25. 10.1108/14777261111161860. 10.1108/14777261111161860 22043650

[73] Person J, Spiva L, Hart P. The culture of an emergency department: an ethnographic study. Int Emerg Nurs. 2013;21:222–7. 10.1016/j.ienj.2012.10.001. 23228617 10.1016/j.ienj.2012.10.001

[74] Arora M, Asha S, Chinnappa J, Diwan AD. Review article: burnout in emergency medicine physicians. Emerg Med Australas. 2013;25:491–5. 10.1111/1742-6723.12135. 24118838 10.1111/1742-6723.12135

[75] Westbrook JI, Raban MZ, Walter SR, Douglas H. Task errors by emergency physicians are associated with interruptions, multitasking, fatigue and working memory capacity: a prospective, direct observation study. BMJ Qual Saf. 2018;27:655–63. 10.1136/bmjqs-2017-007333. 29317463 10.1136/bmjqs-2017-007333 PMC6204927

[76] Kirk JW, Nilsen P. Implementing evidence-based practices in an emergency department: contradictions exposed when prioritising a flow culture. J Clin Nurs. 2016;25:555–65. 10.1111/jocn.13092. 26818380 10.1111/jocn.13092 PMC4738684

[77] Reader TW, Flin R, Mearns K, Cuthbertson BH. Interdisciplinary communication in the intensive care unit. Br J Anaesth. 2007;98:347–52. 10.1093/bja/ael372. 17272386 10.1093/bja/ael372

[78] Benner PE, Tanner CA, Chesla CA. Expertise in nursing practice: caring, clinical judgment & ethics. 2nd ed. New York: Springer Pub; 2009.

[79] Benner P. *Educating nurses: a call for radical transformation* —how far have we come? J Nurs Educ. 2012;51:183–4. 10.3928/01484834-20120402-01. 22476535 10.3928/01484834-20120402-01

[80] Rello J, Afonso E, Lisboa T, Ricart M, Balsera B, Rovira A, et al. A care bundle approach for prevention of ventilator-associated pneumonia. Clin Microbiol Infect. 2013;19:363–9. 10.1111/j.1469-0691.2012.03808.x. 22439889 10.1111/j.1469-0691.2012.03808.x

[81] Gurses AP, Marsteller JA, Ozok A, Xiao Y, Owens AP, Pronovost PJ. Using an interdisciplinary approach to identify factors that affect clinicians’ compliance with evidence-based guidelines. Crit Care Med. 2010;Suppl:S282–91. 10.1097/CCM.0b013e3181e69e02 20647785

[82] Sinuff T, Cook D, Giacomini M, Heyland D, Dodek P. Facilitating clinician adherence to guidelines in the intensive care unit: A multicenter, qualitative study*. Crit Care Med. 2007;35:2083–9. 10.1097/01.ccm.0000281446.15342.74. 17855822 10.1097/01.ccm.0000281446.15342.74

[83] Meier DE. The inner life of physicians and care of the seriously ill. JAMA. 2001;286:3007. 10.1001/jama.286.23.3007. 11743845 10.1001/jama.286.23.3007

[84] Cain CL, Surbone A, Elk R, Kagawa-Singer M. Culture and palliative care: preferences, communication, meaning, and mutual decision making. J Pain Symptom Manage. 2018;55:1408–19. 10.1016/j.jpainsymman.2018.01.007. 29366913 10.1016/j.jpainsymman.2018.01.007

[85] Broom A, Kirby E, Good P, Wootton J, Adams J. The art of letting go: referral to palliative care and its discontents. Soc Sci Med. 2013;78:9–16. 10.1016/j.socscimed.2012.11.008. 23219848 10.1016/j.socscimed.2012.11.008

[86] Dakka FJ. Nurses barriers to evidence-based practice in palliative care: a systematic review. SAGE Open Nurs. 2022;8:23779608221142957. 10.1177/23779608221142957. 36518631 10.1177/23779608221142957 PMC9742693

[87] Ferrell BR, Twaddle ML, Melnick A, Meier DE. National Consensus Project Clinical Practice Guidelines for Quality Palliative Care Guidelines, 4th Edition. J Palliat Med. 2018;21:1684–9. 10.1089/jpm.2018.0431. 30179523 10.1089/jpm.2018.0431

[88] Hartley S, Raphael J, Lovell K, Berry K. Effective nurse–patient relationships in mental health care: a systematic review of interventions to improve the therapeutic alliance. Int J Nurs Stud. 2020;102:103490. 10.1016/j.ijnurstu.2019.103490. 31862531 10.1016/j.ijnurstu.2019.103490 PMC7026691

[89] Rapisarda F, Miglioretti M. Professional culture of mental health services workers: a meta-synthesis of current literature. J Psychosoc Rehabil Ment Health. 2019;6:25–41. 10.1007/s40737-018-0132-2.

[90] Wiltsey Stirman S, Kimberly J, Cook N, Calloway A, Castro F, Charns M. The sustainability of new programs and innovations: a review of the empirical literature and recommendations for future research. Implement Sci. 2012;7:17. 10.1186/1748-5908-7-17. 22417162 10.1186/1748-5908-7-17 PMC3317864

[91] Drake RE, Goldman HH, Leff HS, Lehman AF, Dixon L, Mueser KT, et al. Implementing Evidence-Based Practices in Routine Mental Health Service Settings. PS. 2001;52:179–82. 10.1176/appi.ps.52.2.179. 10.1176/appi.ps.52.2.179 11157115

[92] Le PD, Eschliman EL, Grivel MM, Tang J, Cho YG, Yang X, et al. Barriers and facilitators to implementation of evidence-based task-sharing mental health interventions in low- and middle-income countries: a systematic review using implementation science frameworks. Implement Sci. 2022;17:4. 10.1186/s13012-021-01179-z. 35022081 10.1186/s13012-021-01179-z PMC8756725

[93] Kaihlanen A-M, Hietapakka L, Heponiemi T. Increasing cultural awareness: qualitative study of nurses’ perceptions about cultural competence training. BMC Nurs. 2019;18:38. 10.1186/s12912-019-0363-x. 31440116 10.1186/s12912-019-0363-x PMC6704569

[94] Rogers EM. Diffusion of innovations. 3rd ed. New York; London: Free Press; Collier Macmillan; 1983.

[95] Morena AL, Gaias LM, Larkin C. Understanding the role of clinical champions and their impact on clinician behavior change: the need for causal pathway mechanisms. FrontHealth Serv. 2022;2:896885. 10.3389/frhs.2022.896885. 10.3389/frhs.2022.896885 PMC10012807 36925794

[96] Goedken CC, Livorsi DJ, Sauder M, Vander Weg MW, Chasco EE, Chang N-C, et al. The role as a champion is to not only monitor but to speak out and to educate”: the contradictory roles of hand hygiene champions. Implement Sci. 2019;14:110. 10.1186/s13012-019-0943-x. 31870453 10.1186/s13012-019-0943-x PMC6929350

[97] Martin JB. The integration of neurology, psychiatry, and neuroscience in the 21st century. Am J Psychiatry. 2002;159:695–704. 11986119 10.1176/appi.ajp.159.5.695

[98] Hasse C. Postphenomenology: learning cultural perception in science. Hum Stud. 2008;31:43–61. 10.1007/s10746-007-9075-4.

[99] Conner ML, Clawson JG, editors. Creating a learning culture: Strategy, technology, and practice. Cambridge, UK; New York: Cambridge University Press; 2004.

[100] Beswick C, Bishop D, Geraghty J. Building a culture of innovation: a practical framework for placing innovation at the core of your business. London ; Philadelphia: Kogan Page; 2016.

[101] waterson P, editor. Patient Safety Culture. Farnham: Ashgate Publishing; 2014.

[102] Ehlers UD. Understanding quality culture. Qual Assur Educ. 2009;17:343–63. 10.1108/09684880910992322.

[103] Pathiranage YL, Jayatilake LVK, Abeysekera R. A literature review on organizational culture towards corporate performance. Int J Manag Account Econ. 2020;7(9):522–544.

[104] May C. Towards a general theory of implementation. Implement Sci. 2013;8:18. 10.1186/1748-5908-8-18. 23406398 10.1186/1748-5908-8-18 PMC3602092

[105] May C, Finch T. Implementing, embedding, and integrating practices: an outline of Normalization Process Theory. Sociology. 2009;43:535–54. 10.1177/0038038509103208.

[106] Ranzani Rigotti A, Mara Zamarioli C, Do Prado PR, Helena Pereira F, Gimenes FRE. Resilience of healthcare systems in the face of COVID-19: an experience report. Rev Esc Enferm USP. 2022;56:e20210210. 10.1590/1980-220x-reeusp-2021-0210en. 35635792 10.1590/1980-220X-REEUSP-2021-0210en PMC10081586

[107] Betto F, Garengo P. A circular pathway for developing resilience in healthcare during pandemics. Int J Prod Econ. 2023;266:109036. 10.1016/j.ijpe.2023.109036.

[108] Zhong L, Lopez D, Pei S, Gao J. Healthcare system resilience and adaptability to pandemic disruptions in the United States. Nat Med. 2024;30:2311–9. 10.1038/s41591-024-03103-6. 38956198 10.1038/s41591-024-03103-6

